# Preliminary Estimations of Insect Mediated Transfers of Mercury and Physiologically Important Fatty Acids from Water to Land

**DOI:** 10.3390/biom10010129

**Published:** 2020-01-13

**Authors:** Sydney Moyo

**Affiliations:** Department of Oceanography and Coastal Sciences, Louisiana State University, Baton Rouge, LA 70803, USA; sydmoyo@gmail.com

**Keywords:** aquatic ecosystems, subsidies, eicosapentaenoic acid, docosahexaenoic acid, food webs

## Abstract

Aquatic insects provide an energy subsidy to riparian food webs. However, most empirical studies have considered the role of subsidies only in terms of magnitude (using biomass measurements) and quality (using physiologically important fatty acids), negating an aspect of subsidies that may affect their impact on recipient food webs: the potential of insects to transport contaminants (e.g., mercury) to terrestrial ecosystems. To this end, I used empirical data to estimate the magnitude of nutrients (using physiologically important fatty acids as a proxy) and contaminants (total mercury (Hg) and methylmercury (MeHg)) exported by insects from rivers and lacustrine systems in each continent. The results reveal that North American rivers may export more physiologically important fatty acids per unit area (93.0 ± 32.6 Kg Km^−2^ year^−1^) than other continents. Owing to the amount of variation in Hg and MeHg, there were no significant differences in MeHg and Hg among continents in lakes (Hg: 1.5 × 10^−4^ to 1.0 × 10^−3^ Kg Km^−2^ year^−1^; MeHg: 7.7 × 10^−5^ to 1.0 × 10^−4^ Kg Km^−2^ year^−1^) and rivers (Hg: 3.2 × 10^−4^ to 1.1 × 10^−3^ Kg Km^−2^ year^−1^; MeHg: 3.3 × 10^−4^ to 8.9 × 10^−4^ Kg Km^−2^ year^−1^), with rivers exporting significantly larger quantities of mercury across all continents than lakes. Globally, insect export of physiologically important fatty acids by insect was estimated to be ~43.9 × 10^6^ Kg year^−1^ while MeHg was ~649.6 Kg year^−1^. The calculated estimates add to the growing body of literature, which suggests that emerging aquatic insects are important in supplying essential nutrients to terrestrial consumers; however, with the increase of pollutants in freshwater systems, emergent aquatic insect may also be sentinels of organic contaminants to terrestrial consumers.

## 1. Introduction

The movement of materials between juxtaposed habitats has received much attention by food web and landscape ecologists in the last four decades (reviewed by Richardson and Sato [1]). Freshwater ecologists have long documented that exogenous organic matter (e.g., terrestrial leaves) fuels rivers via inputs of nutrients and organic matter [2], but in recent decades, the importance of aquatic insect subsidies to riparian predators (e.g., bats; [3]) has been emphasized [4,5,6]. These aquatic subsidies are known to affect the behaviour, productivity, and diversity of riparian predators [7,8].

One such subsidy is in the form of physiologically important fatty acids (eicosapentaenoic acid (EPA; 20:5ω3) and docosahexaenoic acid (DHA; 22:6ω3)), both of which are of fundamental physiological importance to all organisms [5,9] because most consumers do not possess the necessary enzymes to synthesize them in the required quantities, so they must obtain them from their diet. These physiologically important fatty acids are required for the maintenance of cell membrane structure and function [10,11], regulating hormonal processes and preventing cardiovascular diseases [12].

Aquatic insects are one group of organisms known to be key exporters of physiologically important fatty acids to terrestrial systems [13], and because many adult insects do not return to the water [14], they represent a net loss of organic nutrients from the aquatic system, and potential food for consumers in adjacent terrestrial ecosystems. A plethora of studies on fatty acids in aquatic systems generally support the premise that aquatic insects are richer in physiologically important fatty acids [15,16,17] than their terrestrial counterparts [13]. Aquatic insects lay their eggs in freshwaters, where the larvae then develop and accumulate physiologically important fatty acids [18]. Subsequently, owing to their complex life cycles, aquatic insects can effectively transfer physiologically important fatty acids to the terrestrial system when they emerge and fall prey to terrestrial predators [19]. As such, knowledge of fatty acids in food sources and consumers is important both for obtaining basic dietary information on consumers within one habitat and for assessing the nutritional implications of reciprocal fluxes in juxtaposed habitats.

Further to providing critical nutrients to terrestrial consumers, aquatic insects can also supply unwanted contaminants to recipient food webs [20]. One such contaminant is mercury, a metal that has become a global concern because of its toxicity. Specifically, methylmercury (MeHg) is of concern as it concentrates at the base of aquatic food webs (e.g., algae) and is subsequently biomagnified, resulting in high concentrations of MeHg in the tissues of predators (e.g., spiders; [21]). The potential of MeHg to be biomagnified presents a health hazard to aquatic organisms and terrestrial wildlife with trophic linkages to aquatic food webs (e.g., those that consume emergent aquatic insects; [22,23,24]). While many studies have examined the movement of contaminants between habitats (e.g., Du et al. [25]), few studies have concurrently measured the fluxes of contaminants and fatty acids from streams to riparian zones; even though stream contamination is widespread [26].

Great strides have been made by individual researchers on the potential export of fatty acids from water to land (e.g., [13,27]), however, studies looking into the potential export by insects are scanty. Furthermore, our current knowledge of transfer of fatty acids and contaminants extends only to site-specific studies (with many being biased toward the Northern Hemisphere), effectively limiting our ability to understand the global effects of stream-derived contaminants and nutrients across aquatic–terrestrial boundaries.

Through the seminal works of Gladyshev and others [18], the first global estimate of physiologically important fatty acids by emerging insects was estimated to be between 0.1 Kg km^−2^ year^−1^ to as high as 672.2 Kg km^−2^ year^−1^. One would expect that with new studies documenting fatty acids in insects, these estimates may have changed significantly. To date, no global estimates are available for the global estimate of mercury from water to land. To this end, the aim of this study was to build on past works by Gladyshev et al. [18] and estimate the continental and global export of contaminants (methylmercury) and nutrients (physiologically important fatty acids) from freshwater systems to land and to determine the extent of coupling between contaminants and nutrients.

## 2. Material and Methods

### 2.1. Literature Search and Data Extraction

To quantify export of physiologically important fatty acids and mercury (Aim: estimate continental and global export of mercury and physiologically important fatty acids via insects) from freshwater systems to land, I quantified subsidies (using physiologically important fatty acids; DHA + EPA) and the potential export of contaminants (methylmercury and total mercury) from freshwater to terrestrial ecosystems by carrying out an extensive search of the scientific literature. To identify relevant studies, a comprehensive literature search was conducted using papers from scientific databases (Google Scholar©, Scholars Portal© and Thomson Reuters Web of Science©) using the search algorithm: fatty acids OR mercury*AND benthic invert*aquatic insects* OR insect emergence. I also included papers from the first global estimates of insect emergence and fatty acids listed in works by Gladyshev et al. [18]. These initial searches yielded >400 articles published up to October 2019. From this initial set, the final dataset (Table 1, Table 2, Table 3, Table 4 and Table 5) was chosen based on the following criteria: (1) emergence reported in mg m^−2^ year (or comparable units) for the year, (2) fatty acids and mercury were reported in mg g^−1^ and ng g^−1^, respectively (or comparable units e.g., ug g^−1^), for benthic insects, (3) only emergence traps were used to collect emergent insects, (4) studies that did not use allometric equations (length-weight regressions) to estimate the dry weight of emergent insects (e.g., [13]) that may overestimate emergence rates [28], and (5) only studies published in English, were included in literature surveys. Criterion 2 excluded studies that reported fatty acid and mercury data as relative proportions or percentages (%).

In several cases, fatty acid, mercury and emergence data were available for different seasons or from different locations. Within a single location, a grand mean was calculated from the fatty acid data from that location, regardless of season; thus, the values represented the average values for a location. Data from different studies were combined to provide a grand mean for each type of data (fatty acid, methylmercury, total mercury, emergence).

To standardise values with those reported in the broader scientific literature, I ensured that all units were converted to match those reported in the literature by other authors [27].

### 2.2. Calculation of Surface Area

Total surface area (Km^2^) was estimated by calculating areas of lakes and rivers for six of the world’s continents (Africa, Asia, Australia, Europe, North America and South America; Table S1 in Supporting information). I used estimates from the Global Lakes and Wetlands Database (GLWD; [29]), Digital Chart of the World (DCW; [30]), HydroSheds (basins and stream networks; [31]) and HydroK1 (US Geological Survey. [32], empirical data supplied by authors [33]) to calculate the total surface area of lakes and rivers. All Shapefiles (.shp) were visualized and surface areas measured using GRASS GIS [34] and QGIS (version 3.10, [35]). For global estimates of surface areas of lakes and rivers, theoretical calculations from several models in the literature were used (see Supplementary information; Table S2).

Aquatic insects develop and live in only a small portion of aquatic habitats. For instance, over 72% of insects only live in the littoral area of lakes near the shore [36]. Similarly, littoral zones can make up anywhere from 3.4% to 30.3% of the surface area of lakes [36]. As such, I adjusted the measurements of all areas to account for the littoral zone to be between 3.4% to 30.3% (average of 18.6% for all Lakes).

### 2.3. Emergence of Insects

**Figure 1:**
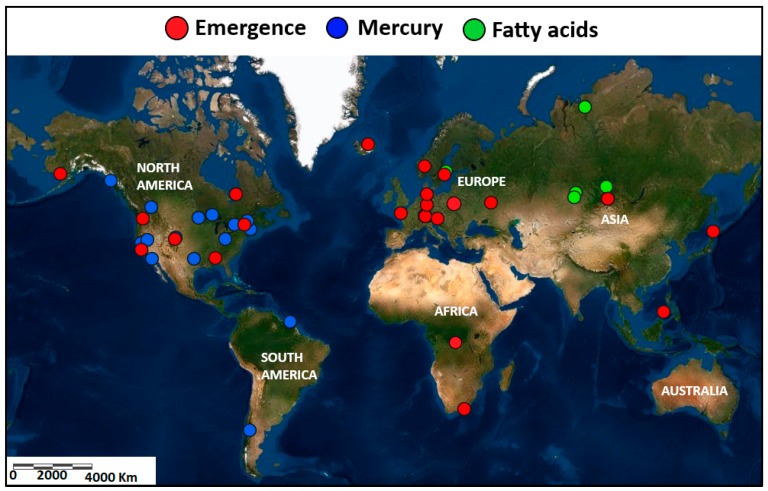
Map showing locality of studies documenting the emergence fatty acids and mercury content in six continents.

Data for emerging aquatic insects (dry weight; g m^−2^ year^−1^) were extracted from diverse literature data (Figure 1; Table 1 and Table 2). Because only a very small percentage of emergent aquatic return to the stream, I used the average calculations of return of insect to freshwaters. For instance, Jackson and Fisher [14] enumerated the return of adult aquatic insects to be only 3.1% of the emerged biomass returned to the stream. Elsewhere, Gray [37] found that less than 1% of aquatic insects in a prairie stream returned to the aquatic system, whereas other researchers have documented larger (9.2%) returns by biomass in lacustrine systems [38,39]. As such, I corrected the net export to account for the return of between 1% to 9.2% for lakes and rivers (average of 4.43% return rate).

### 2.4. Estimates of Physiologically Important Fatty Acids in Aquatic Insects

Available data on physiologically important fatty acids (Figure 1; mg g^−1^ of dry mass) were obtained based on studies that quantitatively determined the fatty acids content of insects using standard fatty acid extraction methods (e.g., [40,41]). Some data reported were for aquatic insect larvae and these were included in the analysed dataset. Fatty acid content of insect differs with life stages from larvae to adults [41], however, the life-stage differences in physiologically fatty acids are minor. For example, some mosquito (Culicidae) larvae and adults have been observed to contain approximately similar quantities of physiologically important fatty acids [41]. Where data were reported as wet weight, I used the moisture content given by the authors to calculate the dry mass. Taxa included were from Europe and Asia (Table 3). Most data collected indicated that Diptera are the most dominant order in most emergence data sets.

### 2.5. Estimates of Mercury and Methylmercury Content in Aquatic Insects

Data on Hg and MeHg (mg g^−1^ of dry mass; Table 4 and Table 5) were obtained based on studies that quantitatively determined the content of the two forms of mercury in aquatic insects using advanced mercury analyzers like amalgamation-thermal atomic absorption spectrometers [48,66]. While original data were presented by most authors in ng g^−1^, I converted the values to mg g^−1^ (by multiplying all ng g^−1^ values by 1 × 10^−6^) for all analyses to match the values reported for emergence data.

### 2.6. Data Analyses

Initially, content for fatty acids (mg g^−1^) was multiplied by the emergence to obtain the export of fatty acids in (Kg Km^−2^ year^−1^). Mercury content data were converted from ug g^−1^ to mg g^−1^ and subsequently multiplied by emergence to obtain methylmercury (MeHg) and total mercury (Hg) as Kg Km^−2^ year^−1^.

To estimate the total net export (Kg year^−1^) of mercury and fatty acids from water to land, export of mercury and fatty acids (Kg km^−2^ year^−1^) were multiplied by the estimate of areas of lakes and rivers (Km^2^) globally and by continent. Because some continents had no available emergence and mercury data for lakes (e.g., Africa, Australia, Asia and South America) and rivers (e.g., South America, I used the grand mean calculated for all available data for each ecosystem type (Lake or River).

All means and coefficients to variations (CV) were calculated for each data type. All mean values for data were compared using MedCalc^®^ (statistical software version 14.8.1, software bvba, Ostend, Belgium; http://www.medcalc.org; 2018) and following procedures described in Altman [71].

## 3. Results

All literature survey data for fatty acids, Hg, MeHg are presented in Table 1, Table 2, Table 3, Table 4 and Table 5. Overall, the data, as evidenced by high coefficients of variation depict that there is a lot of variation in fatty acid and mercury data recorded in the literature. For example, Hg (Table 5) has a coefficient of variation of over 100 percent. Similarly, the grand means for fatty acids and mercury also show large variations across datasets.

### 3.1. Continental Exports of Physiologically Important Fatty Acids

**Figure 2:**
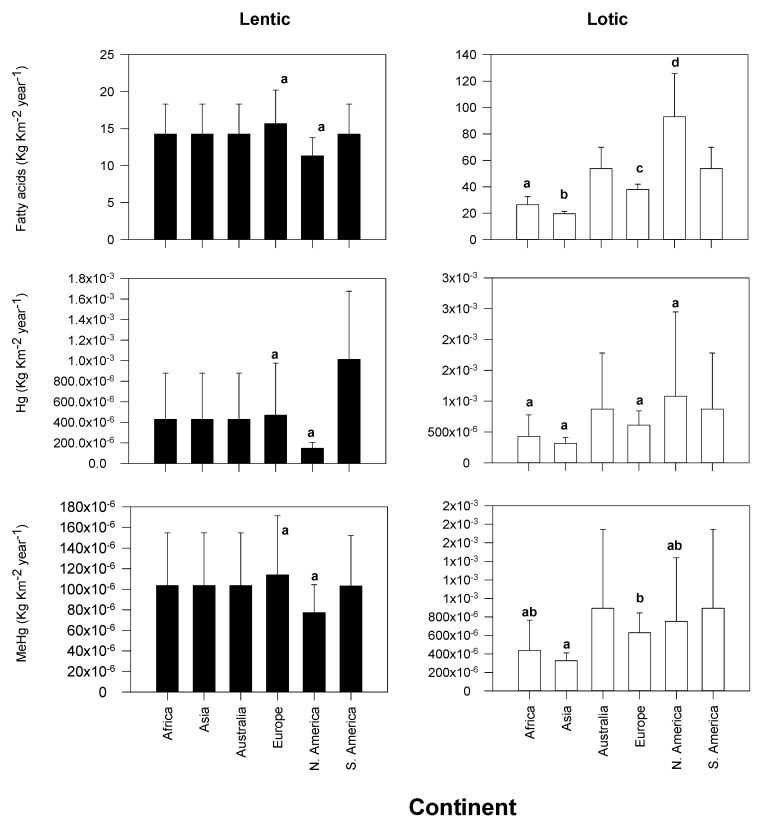
Estimate (±SD) of physiologically important fatty acids, methylmercury (MeHg) and total mercury (Hg) calculated for each continent. The letters depict results from Medcalc^®^ comparison of means calculator within each continent, where values with the same letters depict no significant difference between the export values. Note that only continents where emergence data are available are statistically compared.

Considering export of physiologically important fatty acids per unit area, lentic systems export similar quantities of fatty acids across all six continents in this study (range: 11.3 to 14.2 Kg Km^−2^ year^−1^; Figure 2).

In rivers (Figure 2), North America exports a larger amount of fatty acids (93.0 ± 32.6 Kg Km^−2^ year^−1^; Figure 2) compared to all other continents (range: 19.7 to 53.8 Kg Km^−2^ year^−1^) per unit area. The lowest exports of fatty acids per unit area exported from river to land by aquatic insects were in Asia (19.7 Kg Km^−2^ year^−1^).

**Figure 3:**
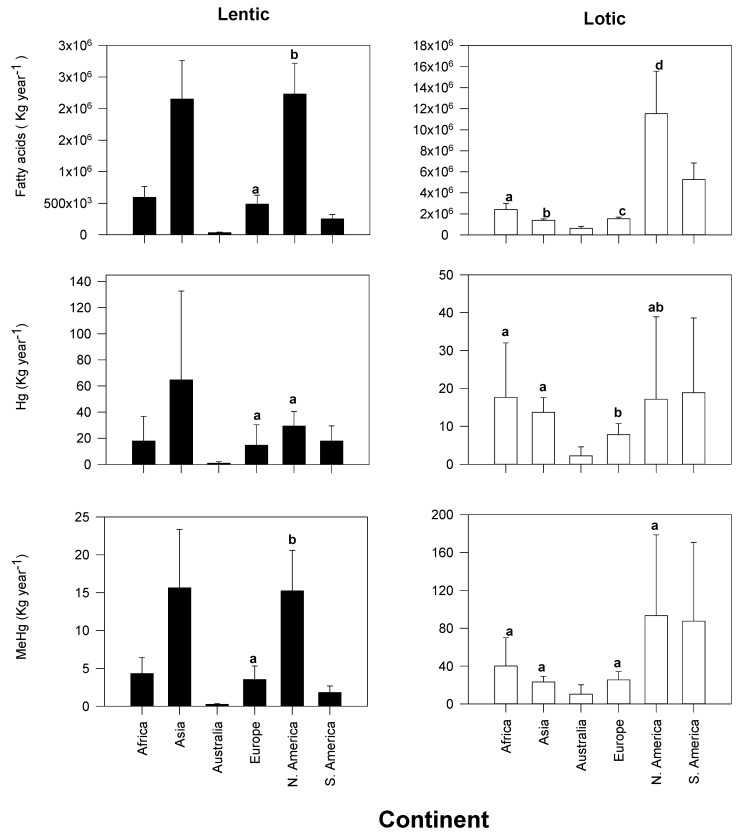
Estimate (±SD) of physiologically important fatty acids, methylmercury (MeHg) and total mercury (Hg) calculated for each continent. The letters depict results from Medcalc^®^ comparison of means calculator within each continent, where values with the same letters depict no significant difference between the export values. Note that only continents were emergence data are available are statistically compared.

Considering the total area of rivers and lakes by continent reveals that the quantity of fatty acids (Kg year^−1^) exported from lakes to land are highest in Asia (2.2 × 10^6^ Kg year^−1^; Figure 3) and North America (2.2 × 10^6^ Kg year^−1^), with Australia exporting the lowest amount of fatty acids (3.4 × 10^4^ Kg year^−1^; Figure 3).

In rivers, North America contributes more to the export of fatty acids (11.5 ×10^6^ Kg year^−1^) than all the other continents (range: 62.4 × 10^4^ to 52.7 × 10^5^ Kg year^−1^). South America is the second largest exporter of fatty acids from river to land (52.7 × 105 Kg year^−1^), with Australia exporting the lowest (62.4 × 10^4^ Kg year^−1^). Overall, rivers across all continents contribute more to export of fatty acids than lakes.

### 3.2. Continental Exports of Mercury and Methylmercury

Regarding the export of Hg and MeHg from lakes to land per unit area, there are no significant differences among the exports of Hg (range: 1.5 × 10^−4^ to 1.0 × 10^−3^ Kg Km^−2^ year^−1^; Figure 2) and MeHg (range: 77.2 × 10^−6^ to 103 × 10^−6^ Kg Km^−2^ year^−1^) in lentic systems.

In rivers, there were no significant differences in flow of Hg from water to land among continents per unit area (mean range: 3.2 × 10^−4^ to 1.1 × 10^−3^ Kg Km^−2^ year^−1^; *p* > 0.05). Similarly, there were no significant differences among exports of MeHg by continent. The only exception was between Europe and Asia, where Europe (6.4 × 10^−4^ Kg Km^−2^ year^−1^) exported more MeHg per unit area from land to water than Asia (3.3 × 10^−4^ Kg Km^−2^ year^−1^).

By considering the total area of rivers and lakes at each continent, I was able to calculate the amount of Hg and MeHg exported from water to land per year (Kg year^−1^). The results from these calculations reveal that there are no significant differences in export of Hg from lakes (Figure 3). Australia was the only exception as it had significantly lower exports of Hg (2 Kg year^−1^) from lake compared to all the other continents. Methylmercury exported from lake to land is greatest in Asia (15.6 Kg year^−1^) and North America (15.2 Kg year^−1^) compared to the other continents (mean range: 0.3 to 4.33 Kg year^−1^).

In rivers, there were no significant difference in exports of Hg and MeHg from river to land, with exceptions occurring between some continents (e.g., export of Hg is significantly higher in Europe than in Australia).

### 3.3. Global Exports of Physiologically Important Fatty Acids and Mercury

**Figure 4:**
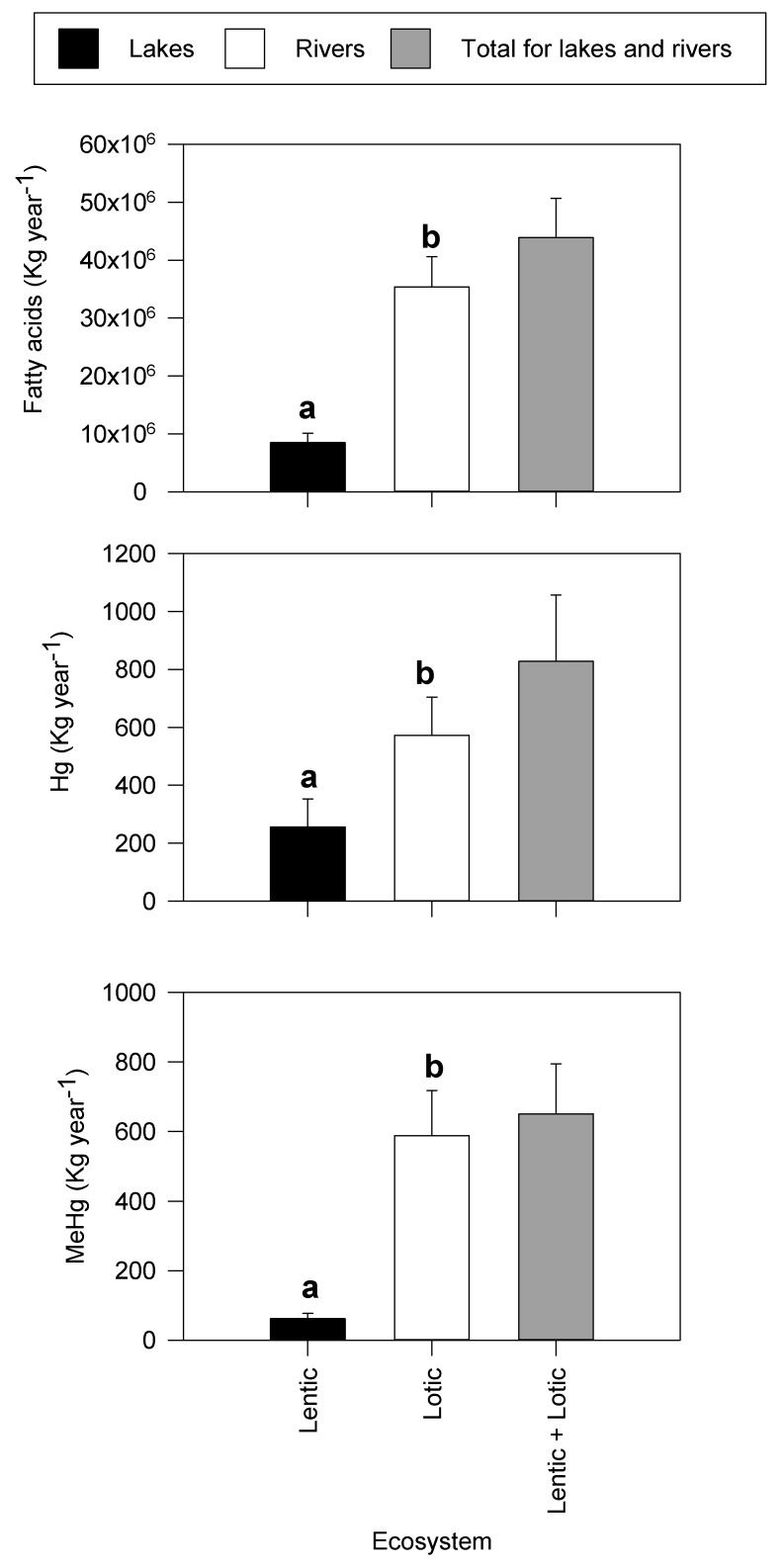
Global estimate (±SD) of physiologically important fatty acids, methylmercury (MeHg) and total mercury (Hg) calculated from diverse ecosystems. The letters depict results from Medcalc^®^ comparison of means calculator between lentic and lotic systems, where values with the same letters depict no significant difference between the export values.

Global export of fatty acids per year are higher in rivers (35.4 × 10^6^ Kg year^−1^) than in lakes (85.1 × 10^5^ Kg year^−1^; Figure 4; *p* < 0.001). Similarly, MeHg exports are higher in rivers (572.1 Kg year^−1^) than in lakes (255.9 Kg year^−1^; Figure 4). Congruent to MeHg exports, Hg differs significantly between rivers and lakes globally (587.7 Kg year^−1^ for rivers versus 61.9 Kg year^−1^ for lakes; Figure 4; *p* < 0.05).

Overall, global estimates reveal that there is some coupling between mercury and fatty acid exports; when fatty export and emergence are high, the values are synchronous to mercury exports by insects (Figure 2, Figure 3 and Figure 4).

## 4. Discussion

Subsidies are known to affect terrestrial consumers in recipient systems, but these cross-boundary fluxes also transport persistent mercury [26]. Here, the first global perspective of the potential synchrony between export of physiologically fatty acids is presented using a plethora of data from different systems. The estimates build on general ideas originally formulated for rivers and lakes as donors of aquatic subsidies via emergent insects [18,72], which have demonstrated the importance of exports of nutrients from water to adjacent land [18,51,72]. One key finding from this this work is that there is synchrony between physiologically important fatty acids and mercury; because of emergence rates. Congruent to previous research (e.g., [54,73]), the results also demonstrate how the export of physiologically important fatty acids and mercury values vary spatially (by continents), with the North American continent exporting more fatty acids from water to land than all other continents.

The estimate of fatty acids exported from water to land (11.3–93.0 Kg km^−2^ year^−1^; Figure 2) are within the range of the first estimate documented to date (0.1 to 672 Kg km^−2^ year^−1^) [18]. The differences in the values obtained may be driven by the availability of more emergence data from other ecosystems. Presently, there are no estimates for export of mercury by aquatic insects to compare with these findings (Figure 1), mainly as a result of prior studies being focused on one aspect on the export of subsidies (nutrients). More studies on the potential export are thus warranted and should yield more fascinating results on the effects of subsidy type on consumers. Considering that hundreds of thousands of miles of streams and lakes are impaired by persistent mercury [74], the results suggest that aquatic insects are likely key movers of mercury from freshwater to terrestrial systems at a global scale. While these estimates are cursory, they may have huge implications for the ecology of terrestrial consumers and humans.

## 5. Implications

### 5.1. Wildlife

**Figure 5:**
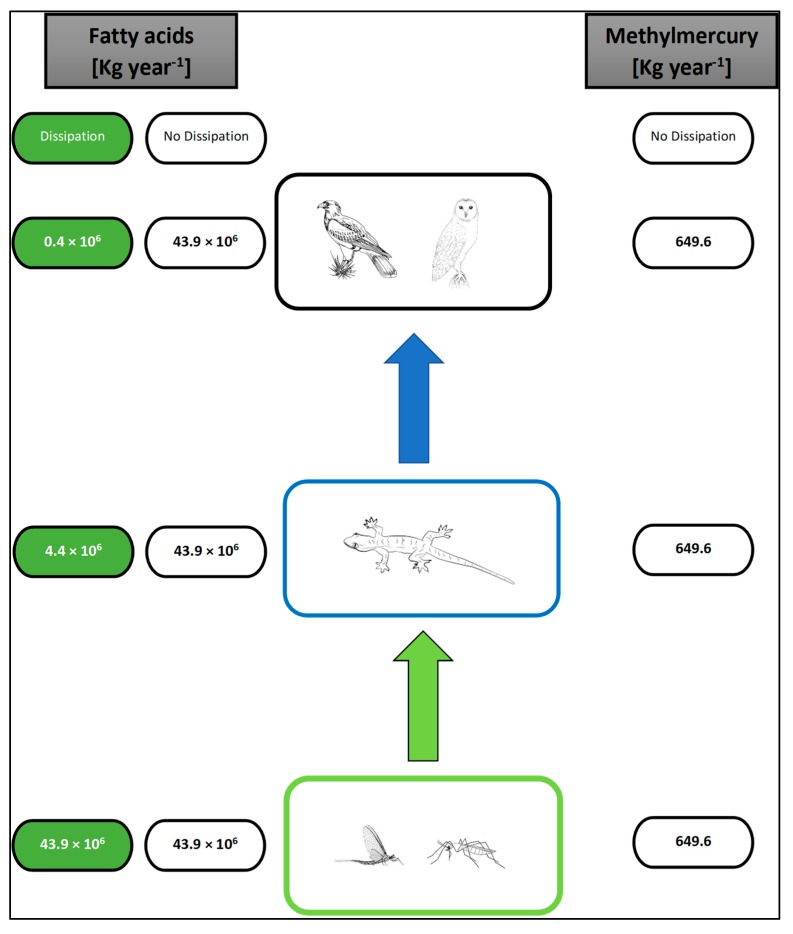
Depiction of the movement of physiologically important fatty acids and methylmercury (MeHg) as mediated my aquatic insects. Transfer efficiency based on traditional trophic pyramid concept of 10% dissipation at each trophic level.

Terrestrial consumers are known to benefit from aquatic subsidies [7]. For example , quality of fatty acids can affect the fitness of tree swallows [75]. Assuming the trophic transfer efficiency of physiologically important fatty acids through the food web to be 10% (i.e., 90% of energy lost at each trophic level; Figure 5) [76,77] in a presumed three-trophic-level food web, aquatic insects can contribute between 0.4 × 10^6^ to 4 × 10^6^ Kg year^−1^ to terrestrial consumers. It is worth noting that while there may be a 10% dissipation with increased trophic level, other researchers have shown that physiologically important fatty acids are retained and are not dissipated by changing trophic positions [78]. To this end, assuming no dissipation of fatty acids happens up the terrestrial food chain implies that fatty acid production of the third level consumers may be equated to the initial contribution of physiologically important fatty acids with insect emergence (Figure 5). The no dissipation scenario is also tenable considering that physiologically important fatty acids are moved through trophic chains at about double the efficiency of biomolecules such as organic carbon and are effectively bioaccumulated (with no dilution) in higher trophic level consumers [79]. However, it must be emphasized that demand by terrestrial consumers for physiological fatty acids is sparse and further studies are warranted to assess terrestrial consumer dietary needs [80].

Terrestrial consumers that depend on aquatic subsidies may suffer irreversible behavioral, physiological, and reproductive effects [81,82] from exposure to MeHg. For example, some birds (e.g., belted kingfisher (*Ceryle alcyon*) and bald eagle (*Haliaeetus leucocephalus*)) and small mammals (e.g., American mink; *Neovison vison*) have been observed to suffer from visual, cognitive, and neurobehavioral effects [82], and even death within a year when exposed to MeHg concentrations of 1 µg g^−1^ [74]. Because MeHg increases in concentration as it progresses up the food chain, one can predict that organisms consuming prey at higher trophic levels are exposed to higher concentrations of total Hg and MeHg (Figure 4; [83,84]). Assuming that MeHg does not change significantly up the food chain suggests that consumers accumulate 649.6 Kg year^−1^. However, the absolute assimilation efficiencies of MeHg vary with trophic level, uptake pathway, and water chemistry conditions; therefore, the estimates need to be interpreted with caution.

### 5.2. Climate Change

Climate warming decreases the production of physiologically important fatty acids by decreasing polyunsaturated fatty acid membrane content while simultaneously increasing saturated fatty acids via homeoviscous adaptation [85]. Specifically, climate warming of 2.5 °C is predicted to reduce physiologically important fatty acid in algae by 8.2% to 27.8% (estimated to reduce physiologically important fatty acids from 240 to 225 tonnes [9]. This reduction under climate change will result in many aquatic insects receiving fewer fatty acids and this may subsequently have major effects on terrestrial consumers that often rely on aquatic subsidies to meet their dietary needs. However, some studies show that temperature does not have an effect on the quantity of physiologically fatty acids in consumers. For instance, Gladyshev et al. [86] found that contrasting temperatures have no effect on physiologically important fatty acids (EPA and DHA) with significant effects only observable in C18 saturated and polyenoic acids. As such, it is plausible that the temperature-dependent decrease in EPA and DHA quantities happens mostly due to changes in the taxonomic composition of aquatic communities as a response to temperature changes [86].

## 6. Additional Considerations and Conclusions

In any study, there are caveats in protocols that can include trap design and other collection tools [87], so some caution is necessary for interpreting any results. I investigated fluxes from river to land using data collected by several authors in different ecosystems, as such, some variation can be expected in these estimates. For example, Different collection methods and traps may overestimate or underestimate fluxes for a variety of reasons [88,89]. Specifically, emergence traps may underestimate the fluxes of odonates from rivers, as some odonates crawl onto vegetation and rocks rather than fly out [87,90]. Additionally, Odonates, individually, have very high biomasses relative to other aquatic insects [90], and their contributions to outward subsidies may be underestimated in all our calculations. I recommend that additional studies incorporate the capture of crawling insects, as this aspect would improve the estimates of aquatic invertebrate flow from water to land.

Additionally, it is worth noting that the values expressed here for annual export of physiologically important fatty acids and MeHg via insects are preliminary estimates, based on averaging data from different ecosystems, and merely represent an initial attempt to calculate the order of magnitude of exports that are mediated my insects. I am cognisant that there are many limitations and sources of error in this type of global extrapolation, including the fact that fatty acids and mercury concentrations may vary depending on region, growth phase, climate, light regime and local nutrient conditions. For example, various authors have shown that mercury varies substantially over space and time [91,92]. Nevertheless, these kinds of data using a global perspective are needed to give a broader scale (*sensu* Gladyshev et al. [18]), which, in the future, may be refined further to create models to predict how environmental perturbations like climate change may affect the spatial and temporal dynamics of subsidies and methylmercury exported from water to land.

Summarily, these results underscore the need to view freshwater systems as just not nutrient exporters but lateral exporters of harmful contaminants [64] that can potentially be biomagnified within the food web. This view departs from the traditional viewpoint of streams being exporters of nutrients alone. Riparian insectivores (e.g., birds and small mammals) facilitate the transfer of aquatic mercury to higher trophic levels, thus serving as conduits in the dispersal of aquatic contaminants to the broader terrestrial food web [82]. Given the widespread contamination of streams, the ubiquity of stream insects, and the importance of insect subsidies to riparian predators, more research is needed to quantify the magnitude and risk of exposure to riparian food webs.

## Figures and Tables

**Table 1:** Insect emergence from lakes (g DM m^−2^ year^−1^) for available continents. ‘Community’ denotes instances where whole taxa values are reported. Average and coefficient of variation in bold represents the grand average that was used to calculate emergence for Africa, South America, Asia, Australia.

Continent	Taxa	Emergence	Reference
Europe			
	Chironomidae, Ephemeroptera, Trichoptera	4.0	[42]
	Community	1.8	[43]
	Community	1.4	[43]
	Community	1.1	[43]
	Community	2.4	[44]
	Chironomidae	1.9	[45]
	Chironomidae	0.2	[46]
	Community	0.2 ^a^	[47]
Average ± SD		1.6 ± 1.2	
Coefficient of variation (%)		70.9	
North America			
	Chironomidae	1.5 ^b^	[38]
	Community	1.1 ^c^	[48]
	Chironomidae	0.2 ^d^	[49]
	Chironomidae	1.9	[14]
Average ± SD		1.2 ± 0.6	
Coefficient of variation (%)		53.8	
**Average ± SD**		**1.5 ± 1.0**	
**Coefficient of variation (%)**		**70**	

^a^ average values calculated from Table 3 of the reference. ^b^ averaged author′s data. ^c^ Recalculated from authors data. ^d^ average value calculated from Table 2 of the reference.

**Table 2:** Insect emergence from rivers (g DM m^−2^ year^−1^) for available continents. ‘Community’ denotes instances where whole taxa values are reported. Values in ‘bold’ denote the grand means and standard deviation for all available data. Average and coefficient of variation in bold represents the grand average that was used to calculate emergence for Australia and South America.

Continent	Taxa	Emergence	Reference
Africa			
	Trichoptera	0.5 ^e^	[14]
	Community	4.0 ^e^	[14]
Average ± SD		2.2 ± 1.7	
Coefficient of variation (%)		78.6	
Asia			
	Community	2.1 ^f^	[50]
	Community	1.2 ^g^	[51]
Average		1.7 ± 0.5	
Coefficient of variation (%)		27.3	
Europe			
	Diptera, Trichoptera, Ephemeroptera	1.7	[52]
	Ephemeroptera, Plecoptera, Trichoptera	3.6 ^h^	[14]
	Ephemeroptera, Plecoptera, Trichoptera	5.0 ^h^	[14]
	Community	5.4 ^h^	[14]
	Community	2.6 ^h^	[14]
	Community	2.6 ^h^	[14]
	Community	3.7 ^h^	[14]
	Community	3.7 ^h^	[14]
	Community	2.0 ^h^	[14]
	Community	2.6 ^h^	[14]
	Community	3.2 ^h^	[14]
	Chironomidae	1.9 ^h^	[14]
Average		3.2 ± 1.1	
Coefficient of variation (%)		35.7	
North America			
	Diptera, Chironomidae	1.2 ^i^	[53]
	Trichoptera, Ephemeroptera, Plecoptera, Diptera	6.6 ^j^	[54]
	Ephemeroptera, Plecoptera, Trichoptera	0.3	[39]
	Chironomidae, Ephemeroptera, Trichopetra	23.1 ^h^	[14]
	Community	5.3	[14]
	Community	7.1	[14]
Average ± SD		7.8 ± 9.2	
Coefficient of variation (%)		117.4	
Average ± SD		**4.5 ± 4.5**	
Coefficient of variation (%)		**100.4**	

^e^ data for Democratic republic of Congo (formerly Zaire) stream from Table 5 of the reference; ^f^ averaged from using average weight of insect specimen dry mass 150 μg; ^g^ recalculated from Figure 1C of the reference; ^h^ data for Europe from Table 5 of the reference; ^i^ averaged author′s data; ^j^ recalculated from authors data.

**Table 3:** Physiologically important fatty acids (EPA+DHA, mg g^−1^ of dry mass) in emergent aquatic insects in lakes and rivers. Taxa in italics represent fatty acids measured in insect larvae. Average and coefficient of variation in bold represents the grand average that was used to calculate emergence for all six continents.

Continent	Taxa	EPA +DHA	Reference
Lentic			
	Odonata	8.27 ^k^	[55]
	Chironomidae	11.9	[46]
	Community	17.8 ^l^	[56]
	Chironomidae	4.0	[40]
	Chironomidae	7.0	[40]
	Ephemeroptera	11.3	[27]
	Chironomidae	10.1	[57]
	Culicidae	6.77	[41]
Average ± SD		**9.6 ± 3.9**	
Coefficient of variation (%)		**41**	
Lotic			
	*Trichoptera* ^m^	11.6	[58]
	*Ephemeroptera* ^m^	12.8	[58]
	*Chironomidae* ^m^	7.7	[58]
	Chironomidae	18.1	[18]
	Trichoptera	9.4	[27]
Average ± SD		**11.9 ± 3.6**	
Coefficient of variation (%)		**30**	

^k^ converted wet weight to dry weight based on authors data of moisture of ~71.7%; ^l^ average estimated from Figure 3 of the reference; ^m^ dry weight estimated from the reference using moisture contents of 83.8% Trichoptera, Chironomidae 78.0%, Ephemeroptera (80%).

**Table 4:** Total mercury (Hg, mg g^−1^ of dry mass) and methylmercury (MeHg, mg g^−1^) in emergent aquatic insects in lakes. ‘Community’ denotes instances where whole taxa values are reported. Average and coefficient of variation in bold represents the grand average that was used to calculate emergence for Africa, Asia, Australia, Europe.

Continent	Taxa	Total Mercury	Methylmercury	Reference
Lentic				
North America				
	Trichoptera, Diptera	^n^ 4.2 × 10^−4^	^n^ 1.6 × 10^−4^	[48]
	Coleoptera	1.8 × 10^−4^	1.1 × 10^−4^	[59]
	Ephemeroptera	1.3 × 10^−4^	1.4 × 10^−5^	[59]
	Hemiptera	2.6 × 10^−4^	1.2 × 10^−4^	[59]
	Odonata	1.4 × 10^−4^	1.0 × 10^−4^	[59]
	Trichoptera	1.3 × 10^−4^	4.9 × 10^−5^	[59]
	Trichoptera	4.9 × 10^−4^	2.5 × 10^−5^	[60]
	Odonata	1.1 × 10^−4^	5.7 × 10^−5^	[60]
	Ephemeroptera	1.1 × 10^−4^	2.1 × 10^−5^	[60]
	Coleoptera	1.5 × 10^−4^	2.0 × 10^−5^	[60]
	Trichoptera	3.8 × 10^−5^	1.6 × 10^−5^	[60]
	Odonata	7.1 × 10^−5^	4.8 × 10^−5^	[60]
	Ephemeroptera	7.5 × 10^−5^	1.9 × 10^−5^	[60]
	Odonata	9.7 × 10^−5^	1.1 × 10^−4^	[61]
	Ephemeroptera	1.1 × 10^−4^	7.9 × 10^−5^	[61]
	Trichoptera	5.0 × 10^−5^	3.7 × 10^−5^	[61]
	Diptera	6.9 × 10^−5^	3.6 × 10^−5^	[61]
	Odonata	-	1.3 × 10^−4^	[62]
	Diptera	-	7.9 × 10^−5^	[62]
	Trichoptera	-	8.9 × 10^−5^	[62]
Average ± SD		1.3 × 10^−4^ ± 8.9 × 10^−5^	6.6 × 10^−5^ ± 4.3 × 10^−5^	
Coefficient of variation (%)		70	65	
South America				
	Diptera	^o^ 1.3 × 10^−3^	-	[63]
	Ephemeroptera	5.7 × 10^−4^	-	[63]
	Odonata	1.7 × 10^−4^	-	[63]
	Plecoptera	2.0 × 10^−3^	-	[63]
	Trichoptera	3.1 × 10^−4^	-	[63]
	Community	2.0 × 10^−4^	3.4 × 10^−5^	[64]
	Community	2.8 × 10^−4^	1.9 × 10^−4^	[65]
Average ± SD		6.9 × 10^−4^ ± 6.4 × 10^−4^	7.0 × 10^−5^ ± 4.9 × 10^−5^	
Coefficient of variation (%)		93	68	
Average ± SD		**2.9 × 10^−4^ ± 4.4 × 10^−4^**	**7.0 × 10^−5^ ± 4.9 × 10^−5^**	
Coefficient of variation (%)		**150**	**70**	

^n^ mean from data presented in Table 3 in authors data; ^o^ units converted from ug g to mg g^−1^.

**Table 5:** Total mercury (Hg, mg g^−1^ of dry mass) and methylmercury (MeHg, mg g^−1^) in emergent aquatic insects in rivers. ‘Community’ denotes instances where whole taxa values are reported. Average and coefficient of variation (in bold) represents the grand average that was used to calculate emergence for Africa, Asia, Australia, Europe, and South America.

Continent	Taxa	Total Mercury	Methylmercury	Reference
Lotic				
North America				
	Diptera	^p^ 4.5 × 10^−4^	^p^ 2.0 ×10^−4^	[66]
	Ephemeroptera	^q^ 3.4 × 10^−5^	^q^ 1.8 × 10^−5^	[67]
	Trichoptera	5.1 × 10^−5^	*	[68]
	Community	2.7 × 10^−4^	*	[69]
	Ephemeroptera	8.1 × 10^−5^	*	[70]
	Plecoptera	6.1 × 10^−5^	7.3 × 10^−5^
	Diptera	2.0 × 10^−5^	*	[22]
Average ± SD		1.4 × 10^−4^ ± 1.5 × 10^−4^	9.6 × 10^−5^ ± 7.5 × 10^−5^	
Coefficient of variation (%)		108	78	
South America				
	Community	5.7 × 10^−4^	5.0 × 10^−4^	[65]
Average ± SD		**1.9 × 10^−4^ ± 2.0 × 10^−4^**	**2.0 × 10^−4^ ± 1.9 × 10^−4^**	
Coefficient of variation (%)		**104**	**95**	

^p^ based on average from authors data; ^q^ based on means of authors data. * Asterisks denote instance where data were not recorded cited reference.

## Supplementary Materials

The following are available online at https://www.mdpi.com/2218-273X/10/1/129/s1, **Table S1**: Continental estimates of surface areas of lakes and rivers based on shapefiles. All data measured and analysed in qGis, **Table S2**: Global estimates of surface area of lakes and rivers from diverse datasets.
