# Supplementary material for: Preliminary Estimations of Insect Mediated Transfers of Mercury and Physiologically Important Fatty Acids from Water to Land
Source: Biomolecules. 2020 Jan 13;10(1):129. doi: 10.3390/biom10010129 (PMC7023014; doi:10.3390/biom10010129)
Supplement: Supplementary file 1 [file biomolecules-10-00129-s001.pdf]

Table S1: Continental estimates of surface areas of lakes and rivers based on shapefiles. All data measured and analysed in qGis

Continent	**Rivers**	**Lakes**				
		1-10Km^2	10-100Km^2	>100 Km^2	Km^2	Littoral
North America	124000.0	252	213.9	594.1	1060300.0	197215.8
South America	97900.6	18.5	25.3	50.6	94400.0	17558.4
Asia	71014.3	85.9	87.2	638.1	811200.0	150883.2
Europe	40473.0	41.2	44.4	81.0	166600.0	30987.6
Australia	11594.5	2.8	4.9	5.1	12800.0	2380.8
Africa	91389.2	7.9	12.7	203.8	224400.0	41738.4
	436371.6				2369700.0	440764.0

Table S1: Global estimates of surface area of lakes and rivers from diverse datasets

**units**	**lotic**	**lentic**	**reference**
Km^2	662000	*	Downing 2012
Km^2	570000	*	Battin et al. 2008
Km^2	624000	3000000	Raymond et al. 2013
Km^2	*	5000000	Verpoorter et al. 2014
Km^2	773000	*	Allen & Pavelsky 2018
Km^2	*	4200000	Downing et al. 2006
Km^2	*	3900000	McDonald et al. 2012
Km^2	*	3160000	McDonald et al. 2012
Km^2	*	4000000	McDonald et al. 2012

Source Bibliography

ALLEN, G. H. & PAVELSKY, T. M. 2018. Global extent of rivers and streams. *Science (New York, N.Y.)* 361:585–588.

BATTIN, T. J., KAPLAN, L. A., FINDLAY, S., HOPKINSON, C. S., MARTI, E., PACKMAN, A. I., NEWBOLD, J. D. & SABATER, F. 2008. Biophysical controls on organic carbon fluxes in fluvial networks. *Nature Geoscience* 1:95–100.

DOWNING, J. 2012. Global abundance and size distribution of streams and rivers. *Inland Waters* 2:229–236.

DOWNING, J. A., PRAIRIE, Y. T., COLE, J. J., DUARTE, C. M., TRANVIK, L. J., STRIEGL, R. G., MCDOWELL, W. H., KORTELAINEN, P., CARACO, N. F., MELACK, J. M. & MIDDELBURG, J. J. 2006. The global abundance and size distribution of lakes, ponds, and impoundments. *Limnology and Oceanography* 51:2388–2397.

MCDONALD, C. P., ROVER, J. A., STETS, E. G. & STRIEGL, R. G. 2012. The regional abundance and size distribution of lakes and reservoirs in the United States and implications for estimates of global lake extent. *Limnology and Oceanography* 57:597–606.

RAYMOND, P. A., HARTMANN, J., LAUERWALD, R., SOBEK, S., MCDONALD, C., HOOVER, M., BUTMAN, D., STRIEGL, R., MAYORGA, E., HUMBORG, C., KORTELAINEN, P., DÜRR, H., MEYBECK, M., CIAIS, P. & GUTH, P. 2013. Global carbon dioxide emissions from inland waters. *Nature* 503:355–359.

VERPOORTER, C., KUTSER, T., SEEKELL, D. A. & TRANVIK, L. J. 2014. A global inventory of lakes based on high-resolution satellite imagery. *Geophysical Research Letters* 41:6396–6402.
